# Horizontal genetic exchange of chromosomally encoded markers between *Campylobacter jejuni* cells

**DOI:** 10.1371/journal.pone.0241058

**Published:** 2020-10-26

**Authors:** Deepti Pranay Samarth, Young Min Kwon

**Affiliations:** 1 Department of Poultry Science, University of Arkansas, Fayetteville, AR, United States of America; 2 Cell and Molecular Biology Program, University of Arkansas, Fayetteville, AR, United States of America; University of Helsinki, FINLAND

## Abstract

Many epidemiological studies provide us with the evidence of horizontal gene transfer (HGT) contributing to the bacterial genomic diversity that benefits the bacterial populations with increased ability to adapt to the dynamic environments. *Campylobacter jejuni*, a major cause of acute enteritis in the U.S., often linked with severe post-infection neuropathies, has been reported to exhibit a non-clonal population structure and comparatively higher strain-level genetic variation. In this study, we provide evidence of the HGT of chromosomally encoded genetic markers between *C*. *jejuni* cells in the biphasic MH medium. We used two *C*. *jejuni* NCTC-11168 mutants harbouring distinct antibiotic-resistance genes [chloramphenicol (Cm) and kanamycin (Km)] present at two different neutral genomic loci. Cultures of both marker strains were mixed together and incubated for 5 hrs, then plated on MH agar plates supplemented with both antibiotics. The recombinant cells with double antibiotic markers were generated at the frequency of 0.02811 ± 0.0035% of the parental strains. PCR assays using locus-specific primers confirmed that transfer of the antibiotic-resistance genes was through homologous recombination. Also, the addition of chicken cecal content increased the recombination efficiency approximately up to 10-fold as compared to the biphasic MH medium (control) at *P* < 0.05. Furthermore, treating the co-culture with DNase I decreased the available DNA, which in turn significantly reduced recombination efficiency by 99.92% (*P* < 0.05). We used the cell-free supernatant of 16 hrs-culture of Wild-type *C*. *jejuni* as a template for PCR and found DNA sequences from six different genomic regions were easily amplified, indicating the presence of released chromosomal DNA in the culture supernatant. Our findings suggest that HGT in *C*. *jejuni* is facilitated in the chicken gut environment contributing to *in vivo* genomic diversity. Additionally, *C*. *jejuni* might have an active mechanism to release its chromosomal DNA into the extracellular environment, further expediting HGT in *C*. *jejuni* populations.

## Introduction

*Campylobacter jejuni* (*C*. *jejuni*) infections cause significant impacts on food safety and public health worldwide. *C*. *jejuni*, a zoonotic bacterial pathogen and one of the major causes of foodborne enteritis in the developing countries, causes around 2 million campylobacteriosis cases every year in the U.S. [1, 2]. Although *C*. *jejuni*-induced diarrhea is self-limiting [3, 4], the infections are frequently associated with post-infection neuropathies like Guillain-Barré syndrome (GBS) [5, 6] and Miller-Fisher syndrome [7]. GBS, a life-threatening disease has a mortality rate of 3~7% among North American and European populations which is mostly associated with respiratory and cardiovascular complications [8].

Many studies point towards genetic diversity within *C*. *jejuni* populations and among different species of genus *Campylobacter* [9–13]. Different genotypes of *C*. *jejuni* are associated with variations in numerous phenotypic traits including host colonization, invasion, sialyation of lipopolysaccharide and serum resistance [14–16]. Genetic diversity explains the greater ability of a bacterial population to survive in a hostile environment and its better adaptation in host gut colonization. *C*. *jejuni* genomes are rather compact (1.6 to 1.8 Mbp) [17, 18], but genomic contents vary significantly among different strains of *C*. *jejuni* [18–20]. Approximately 1,600 genes are present in a *C*. *jejuni* genome [17], but the number of genes estimated in the pan-genome of *C*. *jejuni* is about 2,600 [12, 21]. The pan-genome is defined as the total number of genes (both core and dispensable genes) present in all strains of a clade [22]. Multiple epidemiological reports indicate towards a complex strain diversity of *C*. *jejuni* [23, 24] and a similar observation was made in *C*. *jejuni* strains isolated from an infected chicken flock [19]. It is important to study and understand the patterns of genetic diversification and its underlying mechanisms in *C*. *jejuni*. The understanding will help us comprehend the epidemiology of *C*. *jejuni*-mediated diseases and therefore ultimately lead to effective control and prevention of *C*. *jejuni* infections [25]. The analysis of infecting *C*. *jejuni* strains using strain-typing methods in a chicken flock is typically complicated as a chicken flock is often infected by different genotypes over time [26].

Horizontal gene transfer (HGT) plays a significant role in incorporating genetic diversity to *C*. *jejuni* genomes [27–29]. There are many molecular epidemiologic studies suggesting HGT among *C*. *jejuni* strains occurs at an extraordinary rate [11, 25, 30]. HGT is often defined as any occurrence of passing inheritable material between organisms, which is asynchronous to the reproduction of the organisms [31, 32]. The dynamically changing genetic composition not only influences the diversity of *C*. *jejuni* in the environment and host but also raises challenges for the scientific community to study *C*. *jejuni* physiology. Immense variation in flagellin gene is clearly revealed from FlaA typing of *C*. *jejuni*, which has been commonly used for epidemiological studies [33–35]. *In vivo* chicken colonization studies [34] have demonstrated that HGT could be a major cause of genetic diversity in *C*. *jejuni*.

The occurrence of HGT is ubiquitous in diverse bacterial genera. In species that are naturally-competent, the extent of HGT is often higher as compared to other bacterial species which are not naturally competent [36]. Consequences of HGT are often deleterious to bacteria, leading to the loss of those bacteria over time. However, HGT events resulting in any selective advantage are prone to spread in the population [37, 38]. Naturally, competent bacteria such as *C*. *jejuni* take up DNA from the environment, which can serve as a source of nutrients to cells. At the same time, these extracellular DNA molecules act as a source of exogenous DNA fragments, which sometimes are integrated into the bacterial chromosome to transfer new genetic information [39]. This process of uptake of DNA from the environment by naturally competent bacteria is termed as natural transformation and is a major step in the HGT process in naturally competent bacteria.

In general, HGT in bacteria is achieved through the following fundamental steps: 1) release of DNA from the donor cells, 2) selection of recipient cells through different barriers that restrict the uptake of DNA, and 3) incorporation of introduced DNA into the chromosome of the recipient cells [38, 40]. There are different ways by which these steps can be executed in bacteria. First, the release of DNA from a donor bacterial cell could happen *via* one of these ways- (i) as naked DNA released out of the cell (which is the key contributor of HGT *via* natural transformation), (ii) through mating-pair formation apparatus (secretion machinery for intercellular DNA transfer) during conjugation [41, 42], (iii) *via* pac sites in the process of general transduction (pac site initiates ‘headful’ packing mechanism resulting dispersion of progeny phage) [43] and (iv) integration of phage into bacterial chromosome (transduction). After the release of DNA, selection of recipient cells is controlled by different mechanisms such as barriers of natural transformation, phage receptor specificity in transduction whereas pilus specificity and surface exclusion in conjugation [44]. Finally, the establishment of foreign DNA in recipient cells is achieved by integration, replication, homologous recombination or illegitimate recombination [38]. Stable integration of the foreign DNA entering the cell is usually one of these ways, including 1) homologous recombination, 2) persistence as an episome if permitted by the natural selection process, 3) integration by transposases or phage integrase, and 4) illegitimate recombination mediated by chance when there is a double-strand break repair [37, 40]. The horizontally transferred DNA transiently present in the bacterial cytoplasm must integrate into the bacterial cells to persist for many generations with the exception of plasmids that succeed in replicating as extrachromosomal DNA [38]. Other than HGT, genotypic variations in bacterial cells take place by multiple other mechanisms like spontaneous point mutations and duplication or deletion of the genetic material [45].

Along with natural transformation, conjugation and transduction are other modes of DNA uptake, which mediate HGT in *C*. *jejuni* and in turn contribute to its genetic diversity [37, 46–50]. HGT in *C*. *jejuni* is the result of one of three mechanisms of DNA uptake. Previously, de Boer et al. [34] have shown bidirectional HGT of genetic material *in vivo* during *C*. *jejuni* infection in the chicken gut. In our study, in contrast, we made an attempt to study HGT among *C*. *jejuni* cells *in vitro*. This study also highlights different factors that could affect the completion of HGT process which includes the final step of HGT (establishment of incoming DNA from donor cells into recipient cells). Notably, we co-cultured two *C*. *jejuni* marker strains in which two different selectable antibiotic-resistance markers were stably inserted into different chromosomal loci. We also studied HGT between *C*. *jejuni* cells in the presence of chicken cecal supernatant, which revealed the potential role of chicken cecal content in providing a favourable environment for HGT in *C*. *jejuni* cells thus boosting its efficiency to adapt in dynamically changing environments.

## Materials and methods

### Bacterial strains

*C*. *jejuni* strains *viz*. *C*. *jejuni* NCTC 11168 [51] and *C*. *jejuni* 81–176 [52] used in this study were generously donated by Dr. Michael Slavik (University of Arkansas, Fayetteville, AR., U.S.A). Both *C*. *jejuni* strains were cultured in MH (Mueller Hinton) agar or broth at 37°C in a microaerophilic condition where the gas composition was O_2_ (5%), CO_2_ (10%) and N_2_ (remaining balance). The strains were stored in MH broth containing 15% glycerol at -80°C. For all the experiments *C*. *jejuni* culture was prepared by recovering the cells from the frozen stock onto MH agar plates with appropriate antibiotics (24 hrs incubation) and passing heavy inoculum from culture plate to 5 ml MH broth supplemented with appropriate antibiotics, followed by incubation for 16 hrs. When necessary, appropriate antibiotics were used at the following concentrations: trimethoprim (10 μg ml^−1^), chloramphenicol (Cm; 6 μg ml^−1^) and kanamycin (Km; 50 μg ml^−1^).

### Construction of marker strains of *C*. *jejuni*

In order to examine the transfer of genetic material between *C*. *jejuni* cells, we constructed two *C*. *jejuni* strains with distinct chromosomal antibiotic markers: chloramphenicol resistance (Cm^R^) and kanamycin (Km^R^) resistance gene. Construction of Cm^R^ marker strain (11168 *hipO*::Cm^R^) involved an overlapping PCR protocol [53], which was performed by amplifying and joining three DNA fragments: 1) Cm^R^ gene (amplified from plasmid pRY112 [54]), 2) 400 bp upstream *hipO* gene flanking region, and 3) 400 bp downstream *hipO* flanking region. Three gel-purified DNA fragments were joined using overlapping PCR creating a Cm^R^ marker cassette (*hipO* upstream flanking region-Cm^R^-*hipO* downstream flanking region). The product of the overlapping PCR reaction was transformed into electrocompetent *C*. *jejuni* cells using electroporation at 2,500 V and plated onto MH agar plate with Cm [55]. The sequence of primers used in the construction of 11168 *hipO*::Cm^R^ is listed in Table 1. Putative *hipO*::Cm^R^ mutants were checked for insertion of Cm^R^ marker cassette at *hipO* gene locus by PCR before making stock. Additionally, natural transformation [55] was performed to transfer the deletion cassette to a fresh background of *C*. *jejuni* strain NCTC 11168 using genomic DNA of the confirmed mutant to eliminate any possible unwanted mutations. The resulting mutant strain in the fresh background was again validated by PCR and Sanger sequencing before the strain was used to make a stock and used for the experiments.

**Table 1:** Oligonucleotides used in this study.

Name	Primer sequence (5’→3’)	Use
Lflk_hipO_f	CAAGCAAGGGGCTAAAATAGG	Primers used to amplify the left flanking region of *hipO* gene
Lflk_hipO_r	TCAATCTATATCACGCAATTAACTTGGAAAGGAACACCGCgcttttagctagggctgcaa	
Cat_f	GCGGTGTTCCTTTCCAAGT	Primers used to amplify the chloramphenicol resistance gene.
Cat_r	CAGTGCGACAAACTGGGATT	
Rflk_hipO_f	AAGACTTGCTGAATAAATAAAATCCCAGTTTGTCGCACTGTTTTTAAAACCCCCACAACG	Primers used to amplify the right flanking region of *hipO* gene.
Rflk_hipO_r	TTCCAATCCAAATCAAACTGC	
Inv-1	ATGGCTCATAACACCCCTTGTATTA	Primer to determine the insertion loci of Tn-5 library mutant.
Inv-2	GAACTTTTGCTGAGTTGAAGGATCA	
KAN-2 FP-1	ACCTACAACAAAGCTCTCATCAACC	Sequencing primer used to sequence insertion loci of Tn-5 mutant.
Kan_in_f	AGGATCAGATCACGCATCTTC	Primers used to amplify the insertion junction of genomic locus and kanamycin marker of recombinant cells.
Kan_in_r	AAGTCCACCCAAAACTGCAC	
Cat_in_f	CAAGCAAGGGGCTAAAATAGG	Primers used to amplify the insertion junction of genomic locus and chloramphenicol marker of recombinant cells.
Cat_in_r	CAGTGCGACAAACTGGGATT	
Primer1-f	AAACTCAACAGCTCCCCAAA	Primers used to amplify the *kpsE* genomic region of *C*.*jejuni*.
Primer1-r	CCAGAAAGCGCAAAATATCC	
Primer2-f	CAGCTTTCTATTGCCCTTGC	Primers used to amplify the *cstII* genomic region of *C*.*jejuni*.
Primer2-r	CGGTCTCATATTCTTGATTTTGG	
Primer3-f	AAAGCCAAGCTACCATTACCAA	Primers used to amplify the *docB* genomic region of *C*.*jejuni*.
Primer3-r	TCAACCCAAACCATGAAAGA	
Primer4-f	TGTTTTGCTATCGCAAGCTG	Primers used to amplify genomic region (Locus tag *CJJ81176_0620* and *CJJ81176_0621*) of *C*.*jejuni*.
Primer4-r	TATCATAGCCGTTGCTGCTG	
Primer5-f	TCAATCAAACGCCTAAGTATGG	Primers used to amplify *the ciaB* genomic region of *C*.*jejuni*.
Primer5-r	ACAACGCGTTCAGGAGAAAG	
Primer6-f	TCGCTCTTCGCATAAAACAA	Primers used to amplify the *motB* genomic region of *C*.*jejuni*.
Primer6-r	CATATTTGCCACCTCAAGCA	

For the Km^R^ marker strain, we selected 10 mutants randomly from the *C*. *jejuni* strain 11168 Tn5 transposon mutant library previously described by Mandal et al. [56] and compared their natural transformation efficiency using genomic DNA of the *C*. *jejuni* Cm^R^ marker strain. One strain was selected as the candidate Km^R^ marker strain for further studies based on its natural transformation efficiency being very similar to that of the wild type strain. We used RATE (Random amplification of transposon ends) protocol [57] to determine the insertion site of the selected Tn5 mutant. RATE protocol is a three-step, single primer PCR protocol which is used to amplify the insertion site, followed by sequencing of RATE products to identify the insertion site. We used the primers Inv-1, Inv-2 and KAN2 FP-1 (Table 1) for the RATE protocol, and the PCR product was sequenced by Sanger sequencing using primer KAN2 FP-1. The sequencing result showed that the Tn5 insertion was in *livH* gene, and thus the strain was designated as 11168*livH*::Km^R^.

### Recombination assay

This assay is used to quantify the number of double antibiotic-resistance mutants after receiving an antibiotic-resistant gene from either parent cell after co-culture with parent cells. It is referred to as recombinant assay for simplicity of understanding and is distinguished from natural transformation experiments where purified DNA is used as the source of DNA. During this assay, we assumed that the incoming genetic material (antibiotic resistance gene) is integrated into recipient cells by one of the mechanisms of DNA integration mentioned in the Introduction section. In addition, the term recombinants here refer to the double antibiotic-resistant mutants obtained after co-culture of the parent cell as a result of the allelic exchange of an antibiotic-resistant gene.

**Fig 1:**
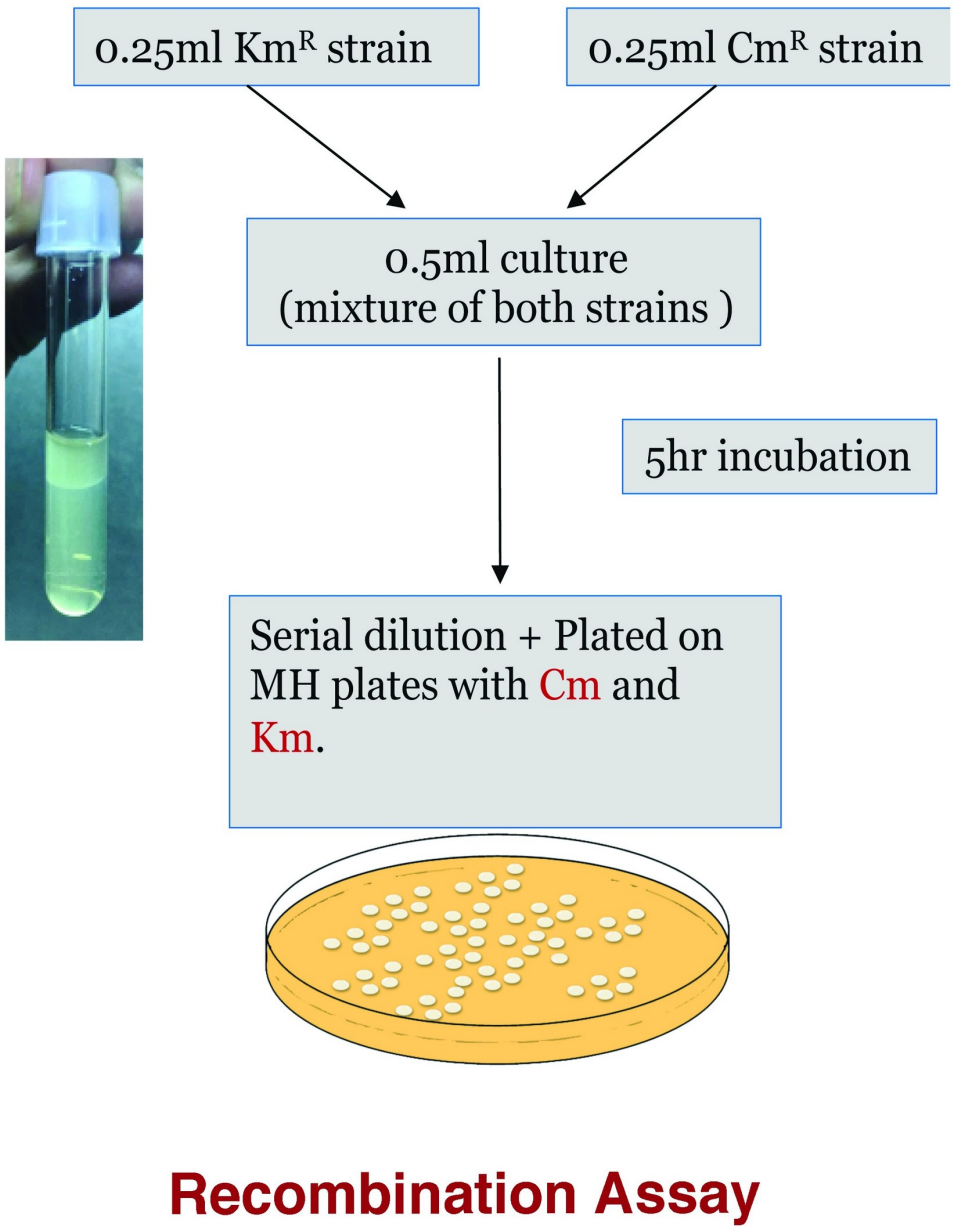
Diagram showing the method used to perform the recombination assay.

Using a heavy inoculum from 24 hrs culture MH plates, both strains (11168 *hipO*::Cm^R^ and 11168 *livH*::Km^R^) was separately suspended in MH broth with appropriate antibiotics and incubated for 16 hrs. Cells were then resuspended in MH broth without antibiotics and diluted to OD_600_ of 0.5 (~1 × 10^9^ CFU/ml) with MH broth. Equal volumes (0.25ml) of both marker strains were mixed together. The final volume of 0.5 ml of the mixed culture was subjected to a biphasic MH system [55, 58] and co-cultured together for 5 hrs at appropriate growth conditions. The number of recombinant cells were determined by CFU of recombinants that were obtained after serial dilutions in phosphate-buffered saline (OmniPur, Millipore Sigma) and were plated onto MH broth agar plate supplemented with both chloramphenicol and kanamycin, followed by incubation for 3 days (Fig 1). The frequency of recombinant colonies obtained as a result of the recombinant assay is also expressed as the percent of recombinant cells obtained out of the total number of both parent strains. This percentage of recovered recombinant cells per recombination assay is hereafter referred to as “recombination efficiency” for simplicity.

### PCR assay to check homologous recombination

**Fig 2:**
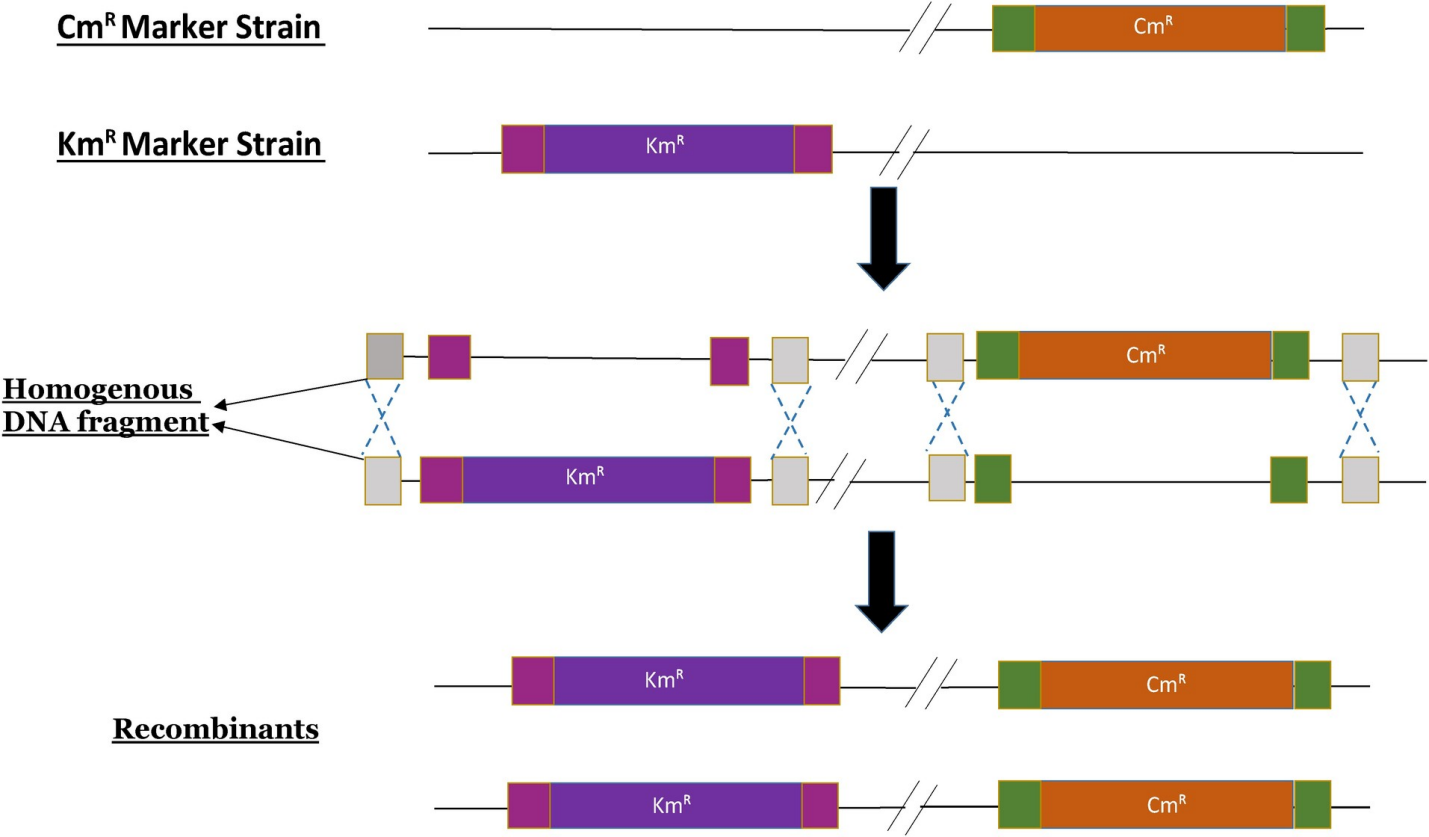
Diagram showing possible homologous recombination that takes place between the two marker strains.

**Fig 3:**
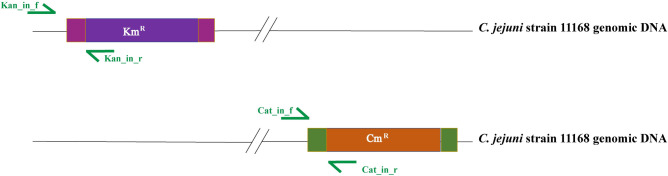
Primer locations used to amplify the insertion junction of antibiotic markers in marker strains.

We hypothesized that the mutants that became resistant to both Cm and Km, are the result of homologous recombination (Fig 2). We designed primers sets (Table 1) corresponding to the insertion junction of DNA markers in such a way that annealing location of forward primer is outside the antibiotic cassette, whereas the reverse primer anneals inside the antibiotic cassette (Fig 3). This way, we would yield amplification only when the antibiotic marker is inserted to the homologous genomic DNA region. We randomly picked 10 double antibiotic-resistant colonies obtained after the recombination assay. We performed colony PCR using these recombinant colonies as DNA templates using the primer sets described above. Along with the recombinant colonies, we also used the parent strains (Cm^R^ and Km^R^; positive control for each locus) and wild type strain (negative control) in the PCR assay.

### Extension of the incubation time to 24 hrs

We aimed to monitor whether the recombination efficiency increased with the longer incubation time of the co-culture (11168 *hipO*::Cm^R^ and 11168 *livH*::Km^R^). We performed the recombinant assay with 24 hrs incubation and then compared the recombination efficiency to that of 5 hrs (control).

### Comparison of recombination in the liquid medium

The recombination assay was also conducted with liquid MH medium in place of biphasic MH medium using essentially the same parameters for the assay. An equal volume of both the marker strains (11168 *hipO*::Cm^R^ and 11168 *livH*::Km^R^) were mixed together and transferred to a 5 ml culture tube. Resulting recombinant colonies were enumerated and the recombination efficiency was compared to that obtained when the biphasic medium (control) was used.

### Recombination across strain background barriers

The marker strains in different strain backgrounds *viz*. 11168 *hipO*::Cm^R,^ 11168 *livH*::Km^R^, 81–176 *hipO*::Cm^R^, 81–176 *livH*::Km^R^ were used to perform the recombination assay in different combinations of the parental strains. We compared the recombination efficiency to examine the role of the potential strain-to-strain barrier in HGT. Natural transformation using genomic DNA of appropriate marker strains was used to construct the markers strains in different strain backgrounds [55].

### Use of cell-free supernatant to evaluate the role of extracellular DNA in HGT

Recombination between *C*. *jejuni* cells can happen after DNA is introduced into the cell *via* multiple pathways including natural transformation. Conjugation is also one of the prominent contributors of the HGT in *C*. *jejuni* cells [37, 59] for which cell-to-cell contact is important. In this experiment, we wanted to determine the frequency of HGT when only the naked, free extracellular DNA present in the supernatant of the *C*. *jejuni* culture is used as a source of DNA for DNA uptake in the recombination assay. After centrifuging for 30 mins, we filtered (0.2 μm filter) overnight culture (OD_600_ of 0.5~1×10^9^ CFU/ml) of marker strains (11168 *hipO*::Cm^R^ and 11168 *livH*::Km^R^) cultured in MH broth. To verify the sterility of the cell-free supernatant, 200 μl of cell-free supernatant was plated on MH agar plate (incubated in microaerophilic condition) and on Luria-Bertani (LB) agar plate (incubated in aerobic condition), followed by incubation at 37°C for 3 days. We performed the recombination assay as described earlier, except that one marker strain was replaced with an equal volume of its cell-free supernatant. The recombination assay where both the marker strains were employed was used as the control for this experiment.

### Recombination experiment in the presence of DNase I treatment

DNase I enzyme nonspecifically cuts DNA and is often used to remove extracellular DNA [60]. We aimed to check the role of extracellular DNA in the present recombination study. Recombination assay between marker strain (11168 *hipO*::Cm^R^ and 11168 *livH*::Km^R^) was conducted in the presence or absence of DNase I. DNase I (NEB) (final concentration of 4 U ml^-1^) was added to the marker strain culture mixture before transferring to a biphasic MH system. After incubation, the number of presumed recombinants were enumerated, and the recombination efficiency was determined as described above.

### Detection of released *C*. *jejuni* chromosomal DNA in the culture supernatant

The cell-free supernatant from a 16 hrs wild-type *C*. *jejuni* 81–176 culture (MH broth) was processed by centrifugation at maximum speed for 30 min, followed by filtration through 0.1-micron syringe filter. The sterility of culture supernatant was checked as described above. We quantified the DNA concentration of the culture supernatant using Qubit^TM^ dsDNA BR assay kit. We performed PCR using six primer sets targeted to amplify different regions of the *C*. *jejuni* genome (Table 1) using a sterile culture supernatant. We used a 2μl cell-free supernatant in 35 cycle PCR. PCR amplification products were visualized using agarose gel electrophoresis.

### Chicken cecal supernatant preparation

The cecal supernatant was prepared by the Lin et al. [61] method with some modifications. Cobb 500 broiler chickens were raised with *ad libitum* access to water and an antibiotic-free corn-soybean meal diet. At the ages between 2 and 3 weeks, 10 birds were sacrificed humanely, and cecal samples were collected aseptically according to the animal use protocol approved by the Institutional Animal Care and Use Committee (IACUC), University of Arkansas. For the collection of cecal samples, chickens of the same age were used to avoid any possible variations due to the ages. The cecal contents were pooled from 10 chickens and mixed with the same volume of MH broth by vortexing, followed by centrifugation at 10,000g (4°C) for 30 min. The supernatant obtained after centrifugation was pre-filtered with different pore size filters (Millipore; 1.2 μm and 0.45 μm filters) to remove all the intestinal tissue debris and microorganisms and finally by 0.2 μm filter. The filtered cecal extracts were tested for sterility as described above.

### Recombination in the presence of chicken cecal supernatant

Different concentrations of sterile chicken cecal extract were added to the MH medium for the recombination assay. Cecal extract media with different concentrations (0.1%, 1%, 10%, 25% and 100%) of cecal extract supernatant were prepared by mixing appropriate volumes of the cecal extract with MH broth to make up the final volume to 0.5 ml. The cecal extract media prepared were used in the recombination assay replacing the MH broth in a biphasic system. The recombinant assay was performed with marker strains 11168 *hipO*::Cm^R^ and 11168 *livH*::Km^R^. The recombination efficiency was determined for each cecal extract medium, and compared with that of the control where biphasic MH media was used for the assay.

### Statistical analyses

JMP genomics software program was used for statistical analysis. The statistical significance of differences between groups was determined by one-way analysis of variance (ANOVA) using by each pair Student’s t-test and *P* < 0.05 was used to determine significant difference. The results were expressed as the mean ± standard error. For comparing 3 or more treatments, All-pair Tukey HSD was used and the difference was considered significant at *P* < 0.05. All experiments were performed at least in triplicate to ensure the replicability of results.

## Results

### Construction of the marker strains

The Cm^R^ and Km^R^ markers were introduced into distinct genomic loci of *C*. *jejuni* strains NCTC 11168, and these marker strains were used for the recombination assay unless described otherwise. To observe the HGT in an experimental setting, it was important to use the marker strains with distinct genomic tags inserted at neutral genomic loci. *hipO* gene locus was used previously by de Boer et. al. [34] to construct a marker strain, which was used to study HGT in *C*. *jejuni* strains. The natural transformation efficiency of 11168 *hipO*::Cm^R^ was comparable to that of the wild type *C*. *jejuni* strain NCTC 11168 (*P>*0.05). One Tn5 mutant strain (Km^R^) with a similar natural transformation efficiency as the wild type *C*. *jejuni* 11168 strain (*P>*0.05) was also selected and used as another marker strain for the recombinant assays. The DNA sequencing of Tn5-flanking regions showed the insertion was in *livH* gene in the Km^R^ marker strain.

### Recombination assay

The recombination experiment was performed to observe the allelic exchange of the antibiotic-resistance markers between the two markers strains (11168 *hipO*::Cm^R^, 11168 *livH*::Km^R^) which are 31kbp apart from each other. The recombination experiment is named as recombinant assay for mere simplicity of understanding and to distinguish it from the natural transformation experiments mentioned in other studies [13, 58, 62, 63] where the purified DNA is used as a source of DNA for the recombination. We believe that double antibiotic-resistant recombinants (mutants) were obtained in the recombination assay as a result of DNA uptake which is carried out by methods such as the natural transformation and conjugation. In the standard recombinant assay, both marker strains were mixed at the ratio of 1:1 in a MH biphasic system and incubated for 5 hrs incubation. The biphasic system is known to enhance the natural transformation efficiency of *C*. *jejuni* [58]. We were able to recover a mean of 1.14×10^4^±0.0571×10^4^ CFUs of the double antibiotic-resistant mutants per recombination assay. When expressed as a percentage of the parental strains, the recombination efficiency was 0.02811 ± 0.0035% of the parent strains.

### PCR validating homologous recombination

**Fig 4:**
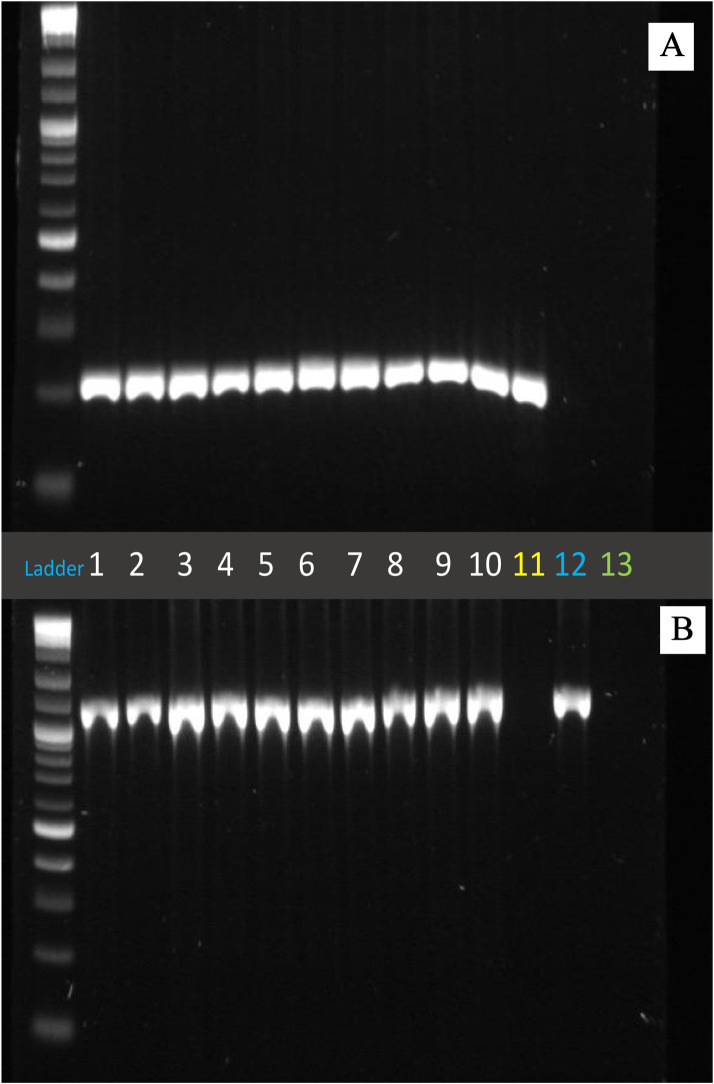
Gel picture showing the result of colony PCR of 10 recombinant colonies picked. A. the result of colony PCR using Kanamycin marker specific primers (Kan_in_f and Kan_in_r). B. the result of colony PCR using Chloramphenicol marker-specific primers (Cat_in_f and Cat_in_r). DNA templates used: Lane 1 to 10- recombinant colonies picked from MH agar plate having kanamycin and chloramphenicol after the recombination assay; Lane 11- parent strain with kanamycin resistance marker; Lane 12- parent strain with chloramphenicol resistance marker; Lane 13- wild-type *C*. *jejuni* strain 11168 with no marker.

Colony PCR with an antibiotic marker specific primer set was performed for 10 randomly selected colonies obtained after the recombination assay on MH agar plate supplemented with both Cm and Km. After running agarose gel electrophoresis (Fig 4), we observed that all 10 colonies used for colony PCR yielded amplification of the products of expected sizes for both primer sets. This result indicates that the antibiotic marker genes were inserted at the same respective loci as in the parent marker strains, suggesting that the HGT was achieved through homologous recombination. On the contrary, the wild type did not yield any amplification, whereas the positive PCR result was obtained only for the corresponding genomic loci for the Cm^R^ and Km^R^ marker strains (Fig 4).

### Extension of incubation time to 24 hrs

**Fig 5:**
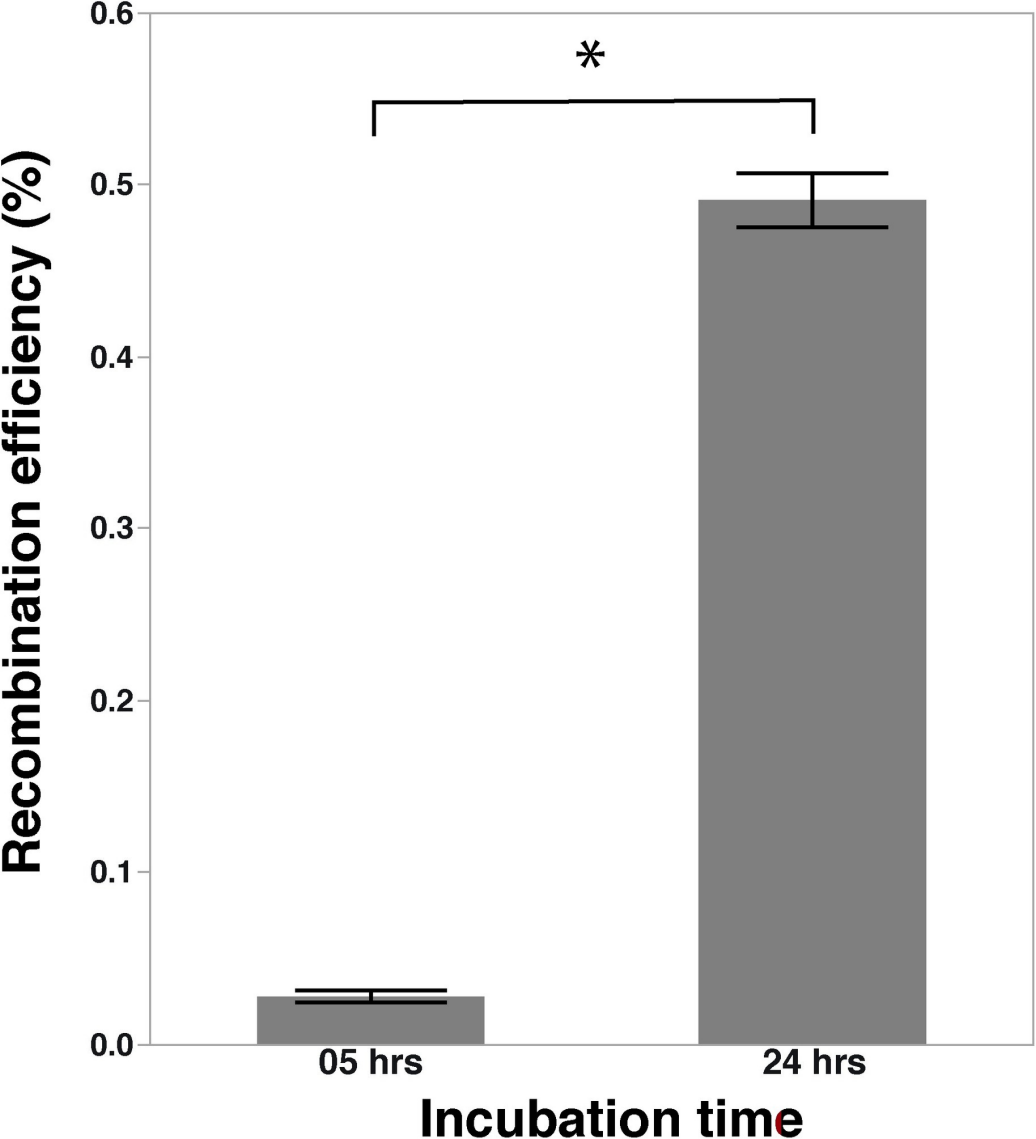
Comparison of the recombinant levels between incubation for 5 hrs (control) vs. 24 hrs. Recombinant levels expressed as percentage recombinants in the total number of parent cells; *indicates a significant difference at *P <* 0.05.

There was an increase in the number of recombinants recovered after 24 hrs as compared to 5 hrs incubation time used in the recombination assay. After 5 hrs incubation (control), we obtained the recombination efficiency of 0.02811 ± 0.0035% of the parent strains and upon longer incubation of 24 hrs, the recombination efficiency was significantly increased to 0.49129 ± 0.01562% of the parent strains (*P* < 0.05) (Fig 5).

### Comparison of recombination in liquid vs. biphasic media

**Fig 6:**
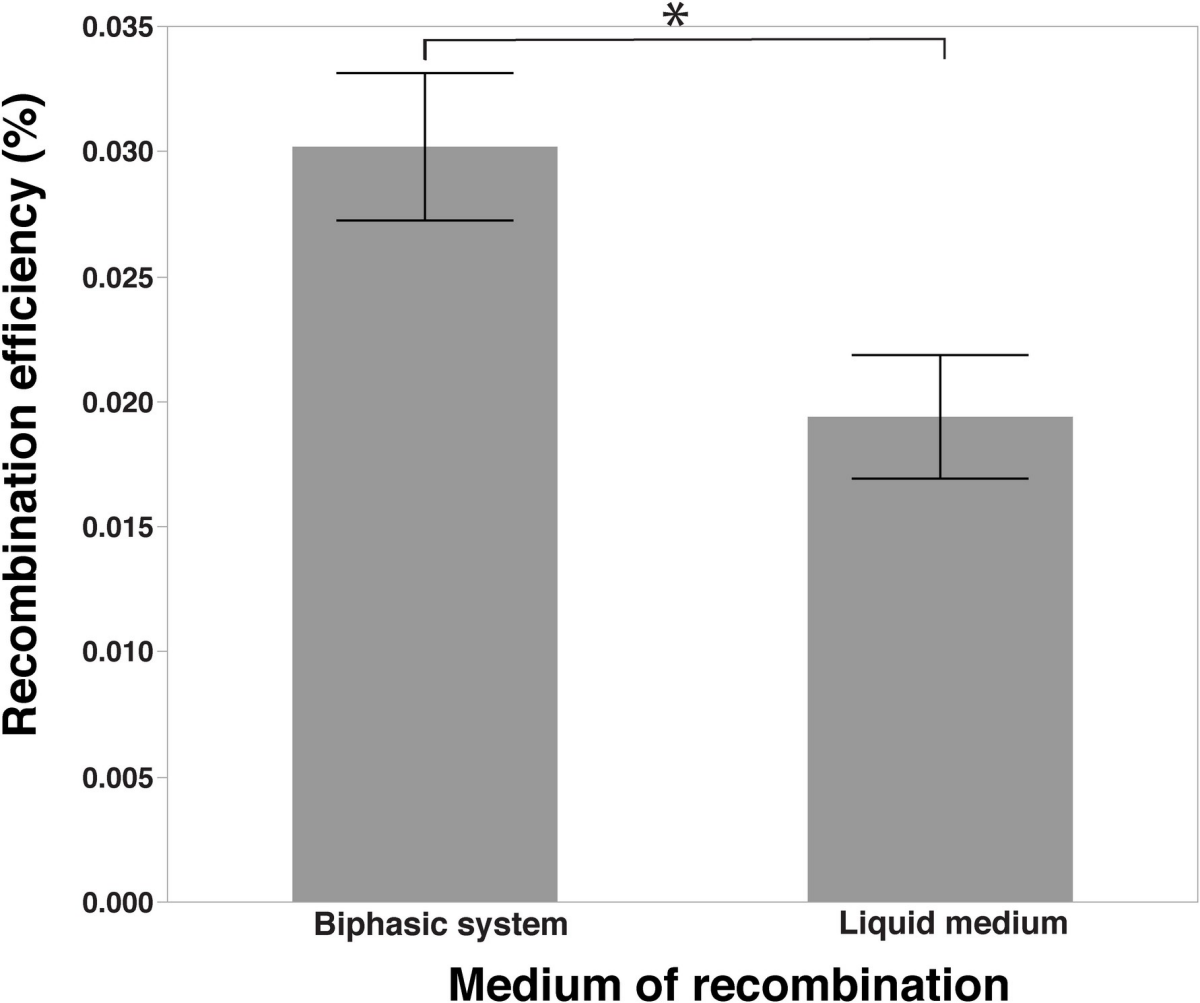
Comparison of the recombinant levels between biphasic medium vs. liquid medium. Recombinant levels expressed as percentage recombinants in the total number of parent cells; *indicates a significant difference at *P <* 0.05.

Biphasic MH system is often considered to have advantages over the liquid medium when performing the natural transformation of *C*. *jejuni* [55, 64]. We obtained the recombination efficiency of 0.0194 ± 0.00246% when MH broth was used to perform the recombination assay, whereas it was 0.03019 ± 0.0029% when MH biphasic medium was used. This result shows that the frequency of recombination event decreased significantly when MH broth was used in comparison to that of MH biphasic medium (*P* < 0.05) (Fig 6).

### Recombination across strain background barrier

**Fig 7:**
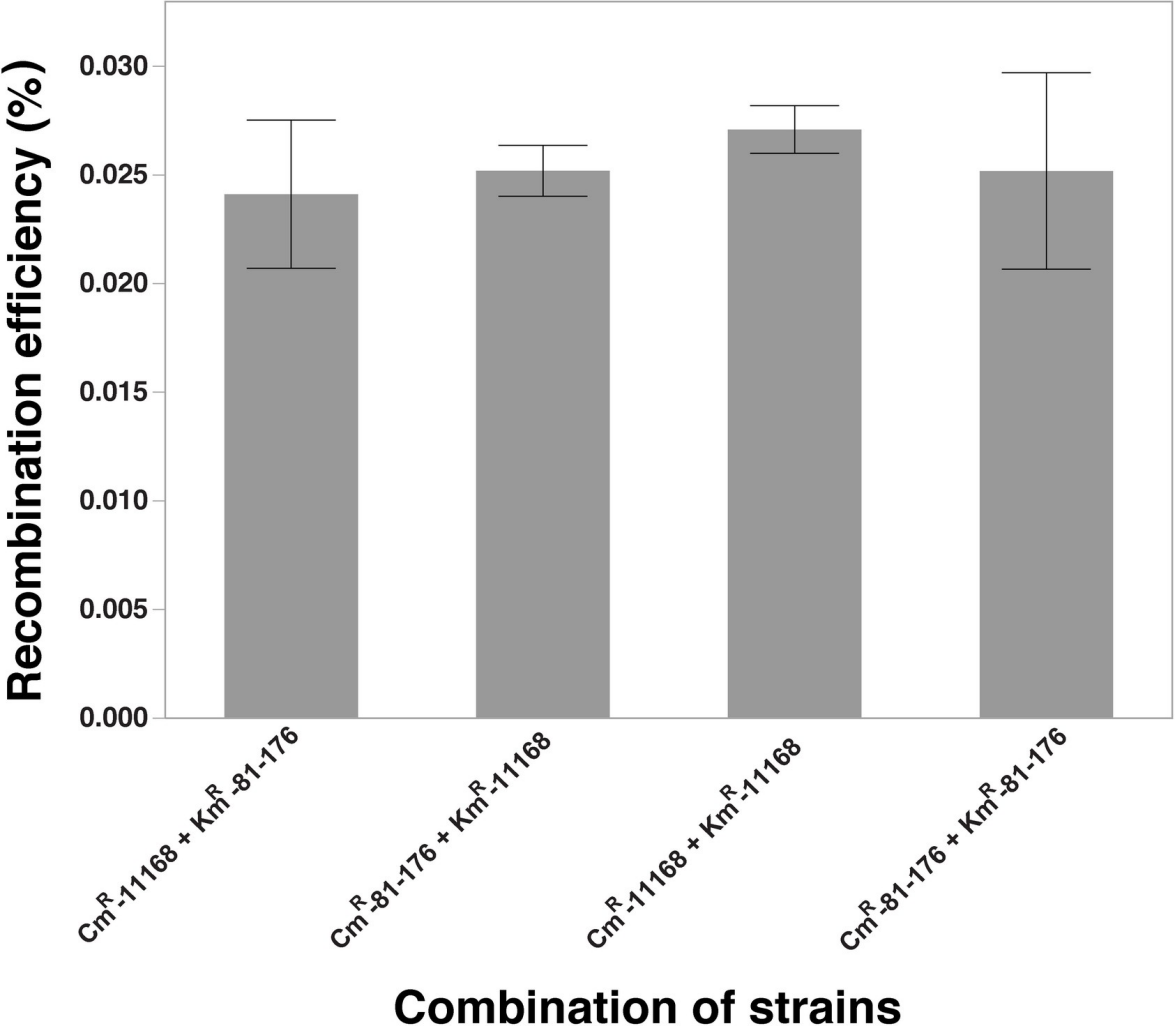
Comparison of the recombinant levels when the different combinations of the marker strains were used. Two different strain backgrounds *(C*. *jejuni* strain 11168 and strain 81–176) were used for different combinations of marker strains; Recombinant levels expressed as percentage recombinants in the total number of parent cells; No significant difference was found across different combinations at *P <* 0.05.

Some studies showed that natural competency differs when different strains of *C*. *jejuni* cells were used during natural transformation experiments [65]. We used the two marker strains in two different strain backgrounds (NCTC 11168 and 81–176) in the recombination assay. The result in Fig 7 shows that the recombination efficiency ranged from 0.0241 to 0.0271% of the parent strains, and there was no significant difference among different combinations of the parent strains (*P* > 0.05).

### Use of cell-free supernatant to evaluate the role of extracellular DNA in HGT

**Fig 8:**
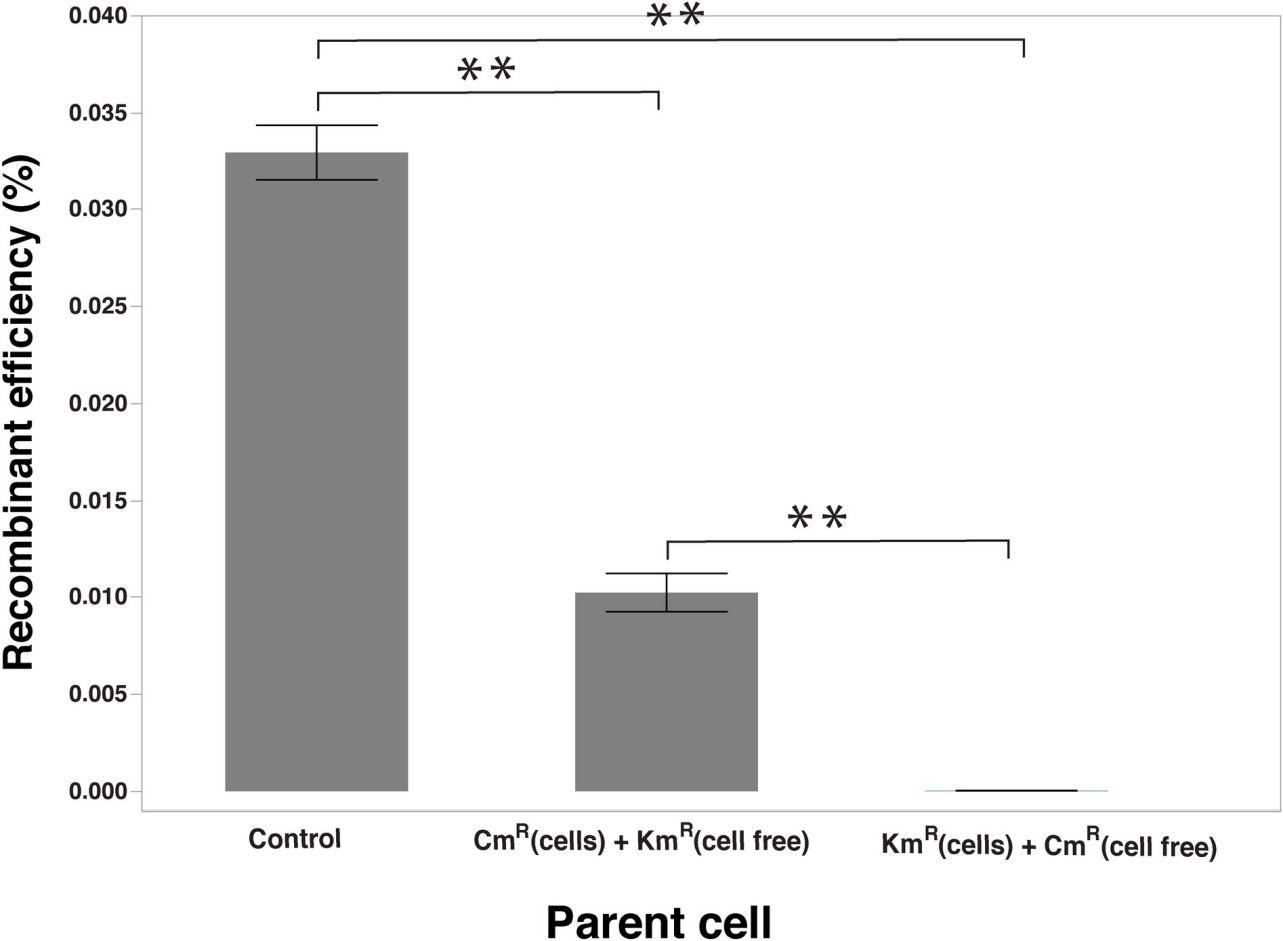
Changes in the recombinant levels when a parent cell is replaced by its cell-free supernatant. The recombinant levels were compared between the standard recombination assay and the modified assay in which one out of two marker cells is replaced by its cell-free supernatant. Recombinant levels expressed as percentage recombinants in the total number of parent cells; **indicates a significant difference at *P <* 0.01.

Cell-free supernatant of the marker strains were prepared by filtering the cell cultures. One of the marker strains was replaced by an equal amount of its cell-free supernatant and then the recombination assay was performed. The number of recovered double antibiotic-resistant cells was compared to that of the control recombination assay where both marker strain cells were used. In the recombination experiment where Cm^R^ (cells) + Km^R^ (cell-free) was used, the number of recovered recombinant cells was significantly lower than the control (0.01024 ± 0.000987% vs. 0.03294 ± 0.0014% of parent strain cells). In recombination experiment where Cm^R^ (cell-free) + Km^R^ (cells) was used, the recombination efficiency reduced more dramatically (0.00002122 ± 0.00001% of the parent strains) (Fig 8). The recombination efficiency with the control recombination assay was 0.0329 ± 0.001405% of the parent strains. This indicates that the absence of cells in the supernatant affects the DNA uptake, which in turn reduced the HGT in *C*. *jejuni* cells. However, the presence of cells is not an absolute requirement for the recombination and DNA transfer can be accomplished by the extracellular DNA.

### Recombination experiment in the presence of DNase I treatment

**Fig 9:**
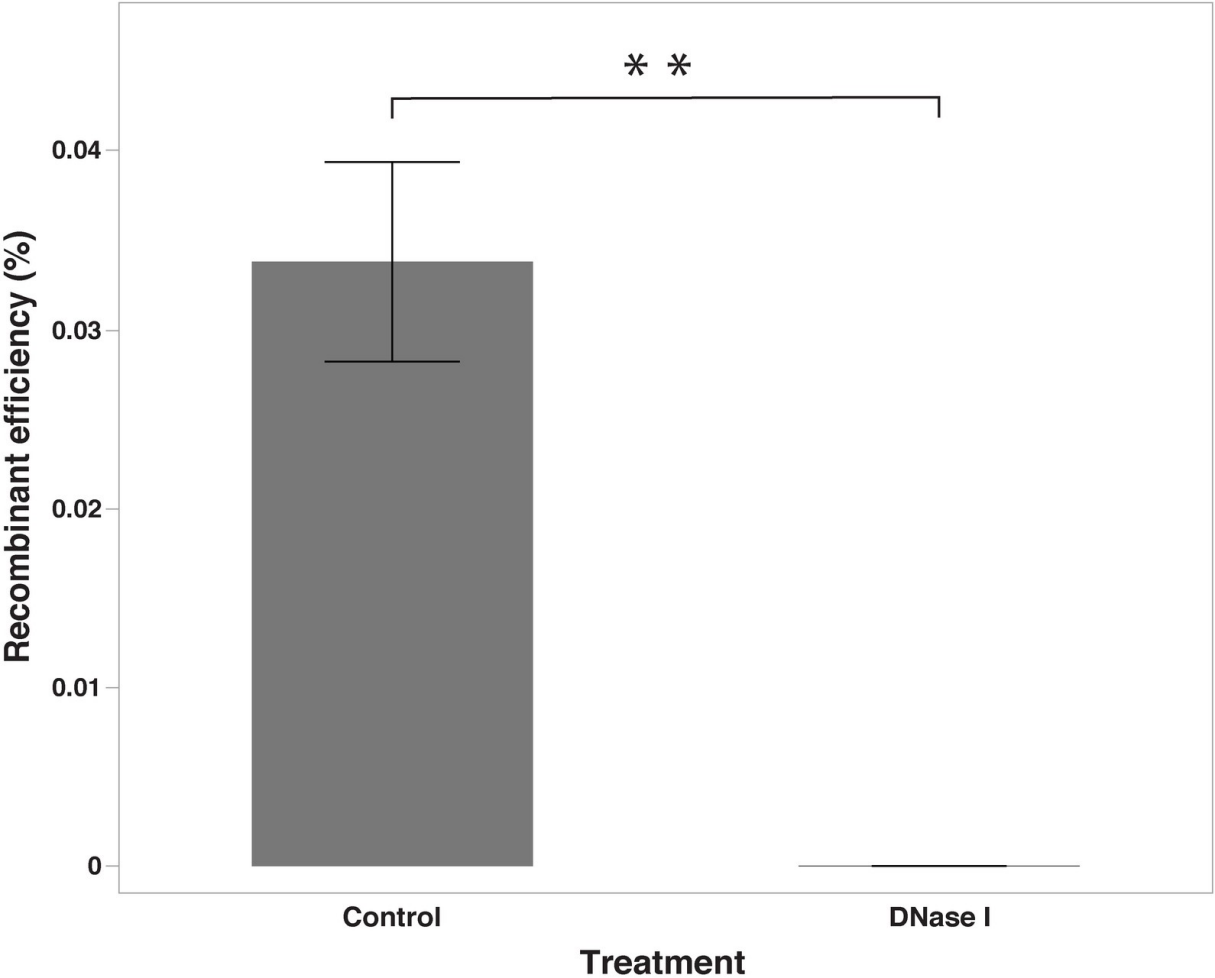
Changes in recombinant levels after addition of DNAse I (DNase I). Recombinant levels were compared when the recombination assay was performed without (control) and with the addition of DNAse I (DNase I) to eliminate extracellular DNA from the medium. Recombinant levels expressed as percentage recombinants in the total number of parent cells; **indicates a significant difference at *P <* 0.01.

We noticed that the number of recombinants recovered after the recombination assay with DNAse I treatment were drastically reduced as compared to the control. There was a decline of 99.92% in the recovery of recombinants when compared to the control (*P* < 0.05). Recombination assay without the DNAse I treatment (control) yielded recombinant efficiency of 0.0338 ± 0.0056% (2.72×10^4^ ± 0.49× 10^4^ CFUs/recombination assay) whereas the recombinant efficiency in the presence of DNase I treatment was 0.0000112 ± 0.0000031% which corresponded to 8.4 ± 2.33 CFUs/recombination assay (Fig 9).

### Detection of released *C*. *jejuni* chromosomal DNA in the culture supernatant

**Fig 10:**
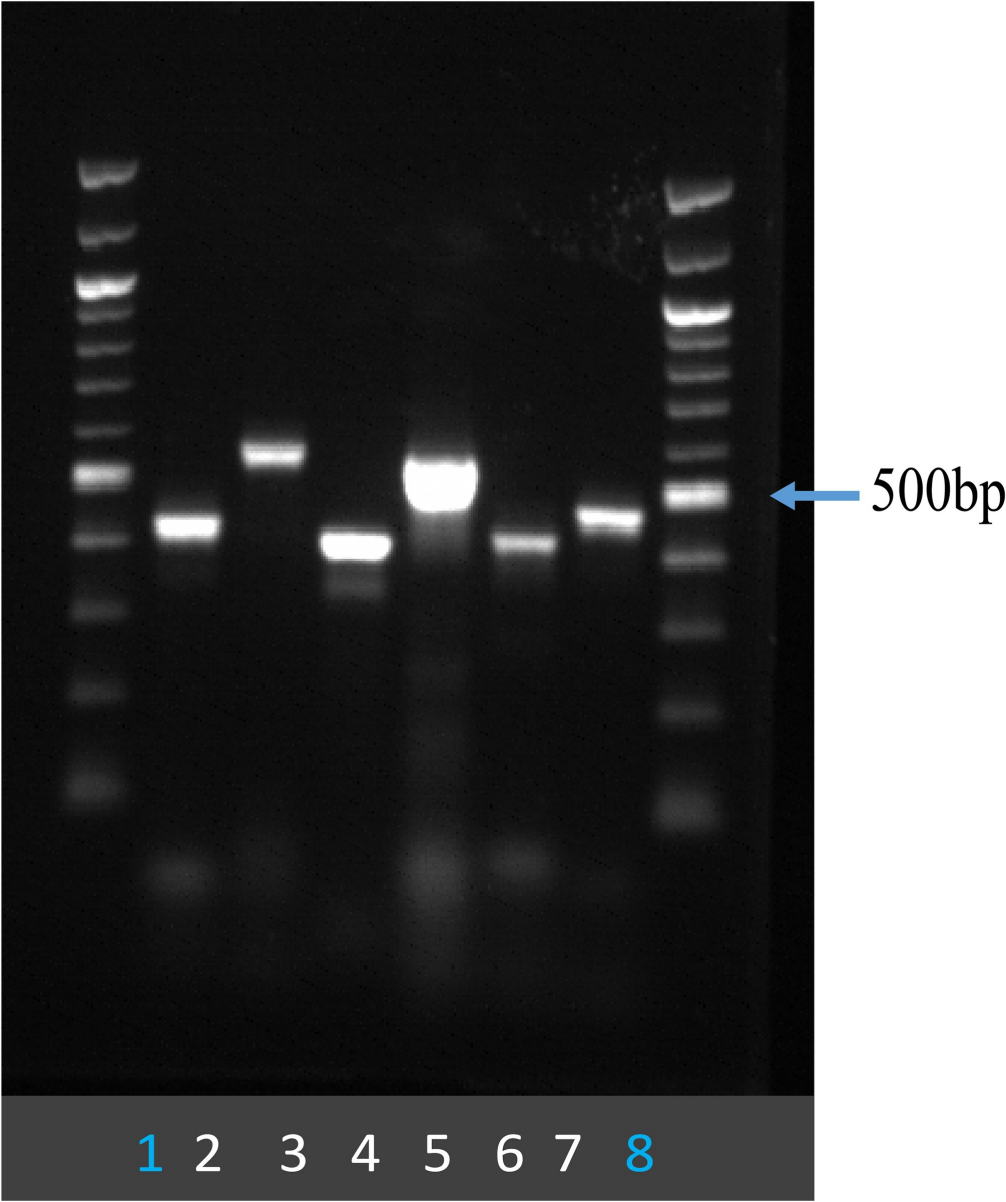
Agarose gel picture showing the result of PCR performed using cell-free culture supernatant. DNA template used in PCR was cell-free culture supernatant of wild type *C*. *jejuni* strain 81–176 with the primer sets targeting six genomic loci; 5 μl of PCR mix was loaded onto the gel. Lane 1 and 8 represent 100kb ladder. Lane 2 to 7 represent the PCR products from *ciaB*, *motAB*, *docB*, *kpsE*, *cstII* and CJJ81176_RS02890 regions, respectively.

High concentrations of extracellular DNA can facilitate HGT in *C*. *jejuni* by increasing encounters of DNA to the recipient cells [66, 67]. Based on the role of extracellular DNA in the HGT events as demonstrated by DNAse I experiment, we wanted to investigate if *C*. *jejuni* can release its chromosomal DNA into the extracellular space. After DNA quantification from three independent experimental replicates, we estimated 2.923 ± 0.13 ng/μl DNA in the cell-free culture supernatant, whereas MH broth alone had a mean of 1.503 ± 0.1 ng/μl DNA. Then, we wanted to check if the DNA of *C*. *jejuni* is present in the extracellular DNA. The culture supernatant from 16 hrs culture of wild type *C*. *jejuni* 81–176 was used as DNA template in PCR reaction using six different primer sets designed to target genomic regions that are positioned with roughly similar intervals throughout the entire *C*. *jejuni* genome (the genomic regions from *ciaB*, *motAB*, *docB*, *kpsE*, *cstII* and *CJJ81176_RS02890*). We used 5 μl of PCR reaction to run agarose gel electrophoresis and found that all six genomic regions were effortlessly amplified from the cell-free culture supernatant, producing the PCR products of the expected sizes (Fig 10). On the contrary, there was no amplification from the supernatant of MH broth alone (S1 Fig).

### Recombination in presence of chicken cecal supernatant

**Fig 11:**
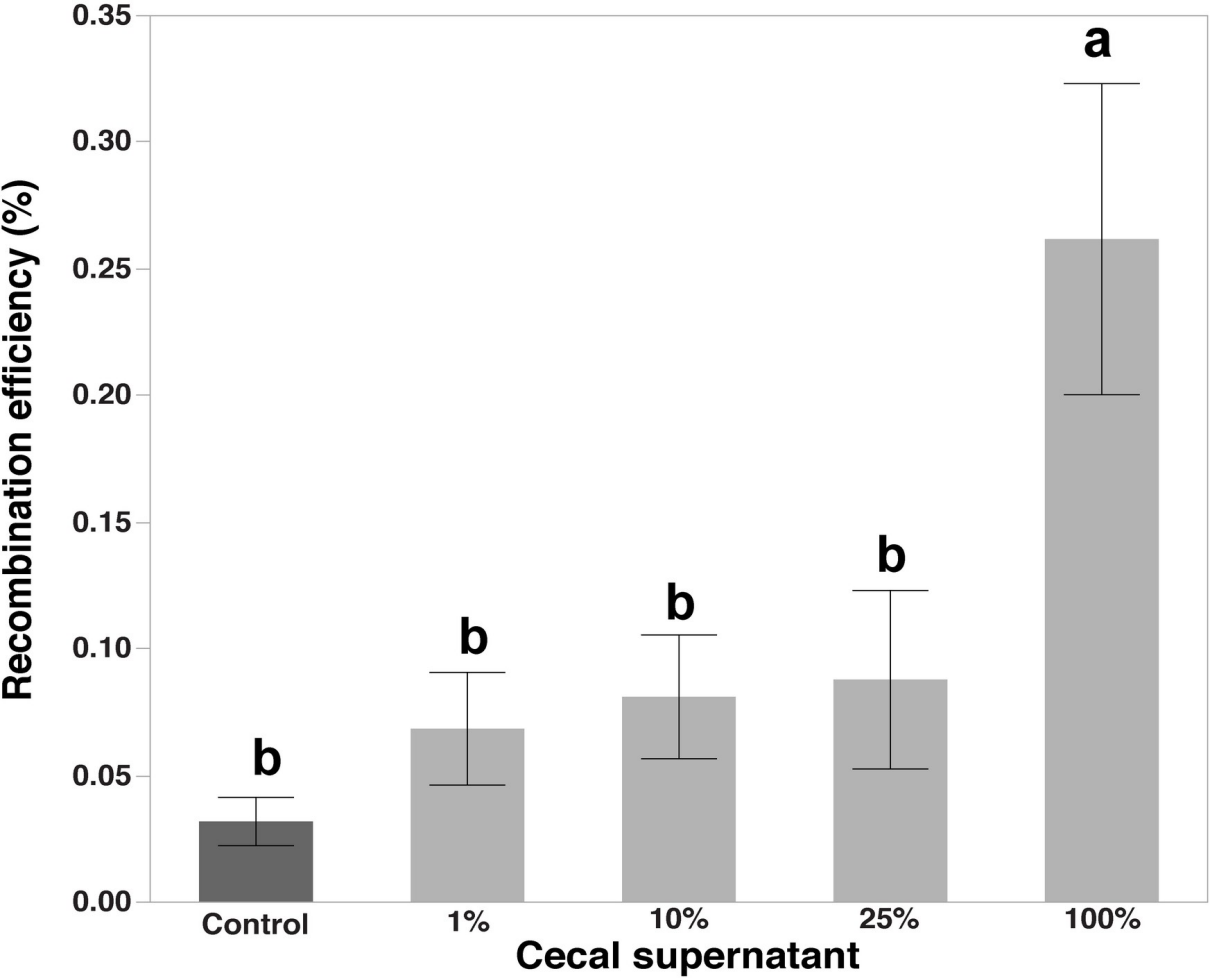
Changes in recombinant levels when the recombination assay performed in the presence of chicken cecal content. The recombination assay was performed in the presence of varying concentrations of chicken cecal contents: Control (0%) and Treatments (1 to 100%). Recombinant levels expressed as percentage recombinants in the total number of parent cells; the bars with different letters are significantly different at *P <* 0.05.

In this experiment, we aimed to study HGT in the presence of a chicken cecal extract. Since the chicken intestinal tract is the main habitat where *C*. *jejuni* colonizes at high levels, we speculated that chicken intestinal environment might facilitate genetic exchanges within the *C*. *jejuni* populations. Cecal content was filtered to remove the cells and tissue debris present in the chicken ceca, retaining only sterile cecal supernatant. For all replicates, fresh cecal contents from chickens at the ages ranging 2~3 weeks were used. We formulated 0.1%, 1%, 10%, 25% and 100% cecal extract medium where MH broth was used to make up the remaining volume. We observed an increasing trend of the recombination efficiency with higher concentrations of the cecal supernatant, although there was no significant difference among the control and 1%, 10% and 25% cecal supernatant media at *P* < 0.05. However, a significant difference (*P* < 0.01) was found with 100% cecal supernatant medium as compared to the control (biphasic MH broth) (Fig 11). The result indicates that HGT through natural transformation in *C*. *jejuni* is facilitated in the presence of cecal supernatant. It might be through increased natural competency of *C*. *jejuni* cells, which is induced by the presence of cecal supernatant. However, it would require additional experiments to evaluate the hypothesis. Another plausible mechanism for increased recombination efficiency is that cecal supernatant induced the lysis of *C*. *jejuni* cells, which in turn increased the recombination efficiency.

## Discussion

It has been well established that *C*. *jejuni* has a high degree of antigenic variation often correlated to the intra/inter-species recombination, helping it escape the host immune surveillance system [63, 68, 69]. In a rapidly changing environment like a chicken intestine, HGT often provides immediate genetic changes, which could be beneficial for adapting to these challenges [38, 70, 71]. Acquisition of antibiotic-resistance genes *via* HGT also contributes to increased fitness of *C*. *jejuni* as demonstrated in multiple studies [72–75]. Thus, cognizance of these important consequences about HGT demands our better understanding of HGT in *C*. *jejuni*.

In this study, we used simple, but convincing *in vitro* experiments to present strong evidence that the HGT between *C*. *jejuni* cells is achieved by homologous recombination mainly using extracellular DNA. We used two marker strains both carrying unique selectable DNA marker (Cm^R^ and Km^R^) at two distinct chromosomal loci for the recombinant assay. After 5 hrs of co-culture of the marker strains, we recovered 0.02811 ± 0.00350% of double antibiotic-resistant recombinants of the parent strains. The recovery of recombinants after co-culture of the two strains of *C*. *jejuni* has been also reported in other studies. Wassenaar et. al. [33] reported the generation of double antibiotic-resistance transformants after co-culture of two *C*. *jejuni* strains [flagellin mutants R1 (*fla*A::Km^R^) and T1 (*flaB*::Tc^R^)]. After 48 hrs incubation, approximately 0.18% recombinants were recovered. Wilson et al. [63] have also observed recombinants after performing co-culture of two *C*. *jejuni* strains (*C*. *jejuni* 81-176-Tn5Cm^R^19 and *C*. *jejuni* 81-176-23SK4) in an experiment based on improved liquid shaking culture to maximize natural transformation efficiency. In a 3 hrs co-cultivation of parent strains, Wilson et al. [63] reported the mean of 0.000188% recovered recombinants. To make the data from our study comparable to those from Wilson et al. [63], we presented our result as the percentage of the parent strains (termed “recombination efficiency”).

We also present the evidence that the DNA markers were exchanged by homologous recombination. The design of marker-specific primers used in our PCR assay ensured that the genetic exchange occurred at homologous loci.

There are numerous studies that point towards frequent occurrences of HGT in *C*. *jejuni* genome. Dingle et al. [24] studied 194 *C*. *jejuni* isolates of diverse origins including humans, animals, and environments using MLST, which suggested a weak clonal population structure where both inter- and intra-species HGT is common. Taboada et al. [76] found that gene variation in *C*. *jejuni* is observed mainly in the variable genomic region and proposed that divergent genes are likely the result of homologous recombination of large genomic fragments. Interestingly, Pearson [10] analyzed genomic diversity between 18 *C*. *jejuni* strains genomes originated from diverse environments and the finding revealed many hypervariable loci clustered together described as hyper-variable plasticity regions. Sheppard et al. [77] speculated that high rate of recombination is causing a reversal of speciation process between *C*. *jejuni* and *C*. *coli*, where it is suspected that *C*. *jejuni* and *C*. *coli* will become one species over the time of microevolution due to the high rate of recombination between them. Both Wassenaar et al. [33] and Harrington et al. [68] have presented strong evidence for inter-genomic recombination between different *C*. *jejuni* strains using polymorphism analysis of PCR-amplified flagellin genes where the start site of the crossover could not be detected as the adjoining DNA sequence was homologous. This suggests a strong possibility of homologous recombination in the flagellin genes. These evidences suggest that gene typing especially using the flagellin gene may not a reliable method to monitor *C*. *jejuni* epidemiology due to frequent inter-species and inter-strain exchange in this genetic locus.

*Escherichia coli*, a model organism to study homologous recombination in Gram-negative bacteria, has at least 25 genetic components involved in homologous recombination [78]. On the contrary, we still have a substantial gap in our knowledge about the mechanism of the homologous recombination in *C*. *jejuni* [79]. RecA is a protein that is known to promote general homogenous recombination in *Campylobacter* spp. [80, 81]. *Helicobacter* is closely related to genus *Campylobacter* and shares some components of the recombination system such as protein AddAB (an enzyme with helicase and exonuclease dual activity). *H*. *pylori* and *C*. *jejuni* RecC protein (an enzyme with both helicase and nuclease activities) shares strong similarity with that of some naturally competent Gram-positive bacteria. Wiesner et al. [82] found that mutation of some *C*. *jejuni* genes which are homologous to components of type II secretion system of Gram-positive bacteria affect DNA uptake (*cts D*, *ctsE*, *ctsF*, *ctsX*, *ctsR*, *ctsP*), recombination and DNA transport across the inner membrane (ctsW, C23) in *C*. *jejuni*. Among 40 known naturally competent bacterial species, *H*. *pylori* and *C*. *jejuni* strain 81–176 share unique machinery for natural transformation, as both require components of type IV secretion system for the natural transformation [82–85]. Significant reduction in transformation efficiency was observed when one of the type IV secretion system genes (*comB3*) was mutated.

Natural competence has been used for genetic manipulation of *C*. *jejuni* for more than a decade [55, 58] and there are enough reports to support that natural transformation plays an important role in DNA uptake facilitating HGT [47, 58, 63, 86]. In this study, we used DNase I enzyme to eliminate extracellular DNA from medium [60, 87]. Our finding from DNase I treatment experiment points the critical role of extracellular DNA present in the medium surrounding *C*. *jejuni* cells in the HGT events observed in our study. This result is in line with Brown et al. [87] and Wilson et al. [63], where they observed a similar reduction in the number of recombinants after DNase I treatment. The extracellular DNA is one of the major drivers of natural transformation [88].

There are different mechanisms by which microorganisms release DNA, including autolysis, active secretion as well as secretion as a part of membrane vesicles. There are reports on the detection of DNA in the culture medium of many naturally competent bacteria such as *Neisseria* spp. [89, 90], *Bacillus subtilis* [91, 92], *Streptococcus pneumoniae* [93] and *C*. *jejuni* [13]. In *N*. *gonorrhea* (a model organism to study natural competence), DNA donation is an active process where DNA is released actively by cell using type IV secretion system (T4SS) and by autolysis [82, 94]. Additionally, in *H*. *pylori*, transcription of a lysozyme-like protein- Lys (HPG27_320) promotes DNA donation from intact cells [95]. *recA* gene of *H*. *pylori* (which promotes general homogenous recombination) is responsible for regulation of both *lys* gene, and the *com* T4SS. A high amount of DNA in the *C*. *jejuni* culture supernatant demonstrated in our current study indicates a similar phenomenon where DNA could be exported to the environment by perhaps a similar mechanism. By regulating competence in a cell density-dependent manner, a bacterium likely becomes competent when it is surrounded by members of its own species and therefore there are a high concentration of homologous donor DNA available for homologous recombination [13, 66, 67, 82, 94]. Dillard and Seifert [89] have reported approximately 0.1–0.2 ng/μl (0.1–0.2 μg/ml) DNA secreted by *N*. *gonorrhoeae* in culture medium (4–24 hrs old culture) which is approximately 5–10 times less than what we reported in this study. Vegge et al. [13] estimated the extracellular DNA present in *C*. *jejuni* strain NCTC 11168 culture-supernatant to be approximately 0.04 μg/ml DNA in early stationary phase. This amount of DNA corresponds to approximately one molecule of a *C*. *jejuni* genome per 100 CFUs and is comparable to that reported for *N*. *meningitides* [90] which actively releases DNA without the cell lysis in the environment. We demonstrated that the amplification of genomic DNA from the cell-free culture supernatant of *C*. *jejuni* is quite straight-forward and result of the PCR assay indicated easy availability of the DNA to surrounding *C*. *jejuni* cells. More research is required in this area to understand the mechanism by which *C*. *jejuni* releases DNA. Svensson et al. [96] have pointed out that no accessory autolysin (lytic transglycosylases) was detected in *C*. *jejuni* which could support lytic mechanism of DNA release. However, they have reported a decrease in CFUs often observed in *C*. *jejuni* cultures after the exponential phase of growth, suggesting possible cell death, leading to the release of DNA. *N*. *gonorrhoeae* uses type IV secretion system for active release of eDNA (extracellular DNA) to the extracellular medium, and it was demonstrated that this secreted DNA is taken up by other cells from the same species or genus contributing towards HGT [97, 98]. We know that till now components of T4SS is not found in *C*. *jejuni* strain 11168, but T4SS is present in many species of genus *Campylobacter* and is possibly involved in its conjugative plasmid transfer or secretion of virulence factors [18]. We speculate that it is possible that *C*. *jejuni* also have a specific mechanism to release DNA without cell lysis, which warrants more attention in the future.

Along with natural transformation, DNA can be transported by other mechanisms in *C*. *jejuni* like conjugation [59, 99] and transduction [100]. Conjugation is one of commonly used technique in laboratories for *C*. *jejuni* genetic manipulation [55, 59, 101, 102]. We studied the changes in the recombination efficiency when cell-free supernatant was used in the recombinant assay replacing the culture of one of the parent strains. Since cell-free supernatant of cell culture contains no cells but free extracellular DNA, it would (i) eliminate the possibility of conjugation that requires cell-to-cell contact and (ii) remove extracellular DNA that might be attached to the cell surface, reducing total extracellular DNA available for natural transformation [103]. Wiesner [67] have previously used cell-free supernatant to demonstrate that *C*. *jejuni* releases DNA to its growth medium, which accelerates the recombination process. We found that the recombination was possible when mere supernatant of overnight marker strain culture was present in the recombinant assay although the overall efficiency of recombination decreased. This suggests an important role of free extracellular DNA in *C*. *jejuni* HGT process and that the absence of cells (which are essential for conjugation) is not a limiting factor for HGT in *C*. *jejuni* cells.

*C*. *jejuni* is naturally competent but is selective in DNA uptake suggested by some reports [63, 65, 104]. While some other Gram-negative bacteria like *N*. *gonorrhoeae* and *H*. *influenzae* identifies self-DNA based on specific DNA sequence repeated in their genome, there is no evidence supporting this possibility for *C*. *jejuni* [104]. However, some reports suggest that *C*. *jejuni* can recognize non-self-DNA based on the DNA-methylation patterns [105]. Beauchamp [104] has described the role of methylation of DNA as a robust system to discriminate non-self-DNA from self-DNA which is regulated by the two-component restriction-modification system that is comprised of paired restriction endonuclease and methyl-transferase. Many *C*. *jejuni* strains contain one Type I RM and four Type II systems [106, 107]. While there are many pieces of evidence supporting the hypothesis that *C*. *jejuni* restricts DNA from other genera/species [47, 58], some suspect the preference towards DNA from the same strain. On the contrary, we found that when the recombination assay was performed using the marker strains in two different backgrounds, the result was not significantly different from that with the same strain combinations. It might be that the host-restriction barrier in *C*. *jejuni* is conserved within the species so that the barrier might be lower or negligible among different strains of *C*. *jejuni*.

Many reports substantiate that HGT in *C*. *jejuni* cells takes place in the environment [108]. Chickens are the natural host of *C*. *jejuni*. *C*. *jejuni* infected chickens carry a very high *C*. *jejuni* load in their gastrointestinal tract, especially in the ceca [109] and considered to have an advantage in colonization as compared to other hosts [110]. Wilson et al. [111] analyzed genetic variation in *C*. *jejuni* cells before and after passage through broilers and mice. *C*. *jejuni* was found to be more adapted with a high frequency of genetic variation and a higher degree of genetic diversity when passaged through broilers as compared to that of mice. The data suggests that the broiler gastrointestinal tract provides an environment that promotes outgrowth and genetic variation in *C*. *jejuni*. The enhancement of genetic diversity in chicken gut may contribute to the fact that chicken gut is the major reservoir of *C*. *jejuni*. Reports have suggested that HGT occurs during colonization in the chicken gut environment [28, 34, 112]. Genomic rearrangement as a result of gene transfer between *C*. *jejuni* and *C*. *coli* strains during colonization was also observed by Korolik et al. [113]. We hypothesize that the chicken gut environment provides suitable condition that facilitates recombination of *C*. *jejuni* cells through specific yet unknown mechanism. In this study, we found that when sterile chicken cecal supernatant was used as a medium for recombination [61], the frequency of recovered recombinants was 10-fold higher in comparison to MH broth. A previous study that reported increased HGT *via* recombination in the presence of sodium deoxycholate supports the hypothesis that bile acid in cecal supernatant could be responsible for increased HGT observed in our study [96]. However, it would require further studies to elucidate the underlying mechanism(s). Increased HGT in the chicken gut environment would enhance genomic diversity within *C*. *jejuni* population in the chicken gut, promoting adaptability as a population to the gut environment, which constantly present challenges. It would be interesting to further expand this research by identifying specific molecules or metabolites responsible for increased HGT in the chicken gut environment. It would reveal a clearer picture of the strategies employed by this pathogen to adapt to the dynamic gut environment.

## Supporting information

S1 Fig Agarose gel picture showing the result of PCR performed using sterile MH broth. (TIF)

S1 Raw images Raw gel picture used in study. (PDF)

## Abbreviations

MH: Mueller-Hinton
LB: Luria-Bertani
Cm: Chloramphenicol
Km: Kanamycin
Cm^R^: Chloramphenicol resistant
Km^R^: Kanamycin resistant
