# Supplementary figures and images for: Horizontal genetic exchange of chromosomally encoded markers between Campylobacter jejuni cells
Source: PLoS One. 2020 Oct 26;15(10):e0241058. doi: 10.1371/journal.pone.0241058 (PMC7588059; doi:10.1371/journal.pone.0241058)

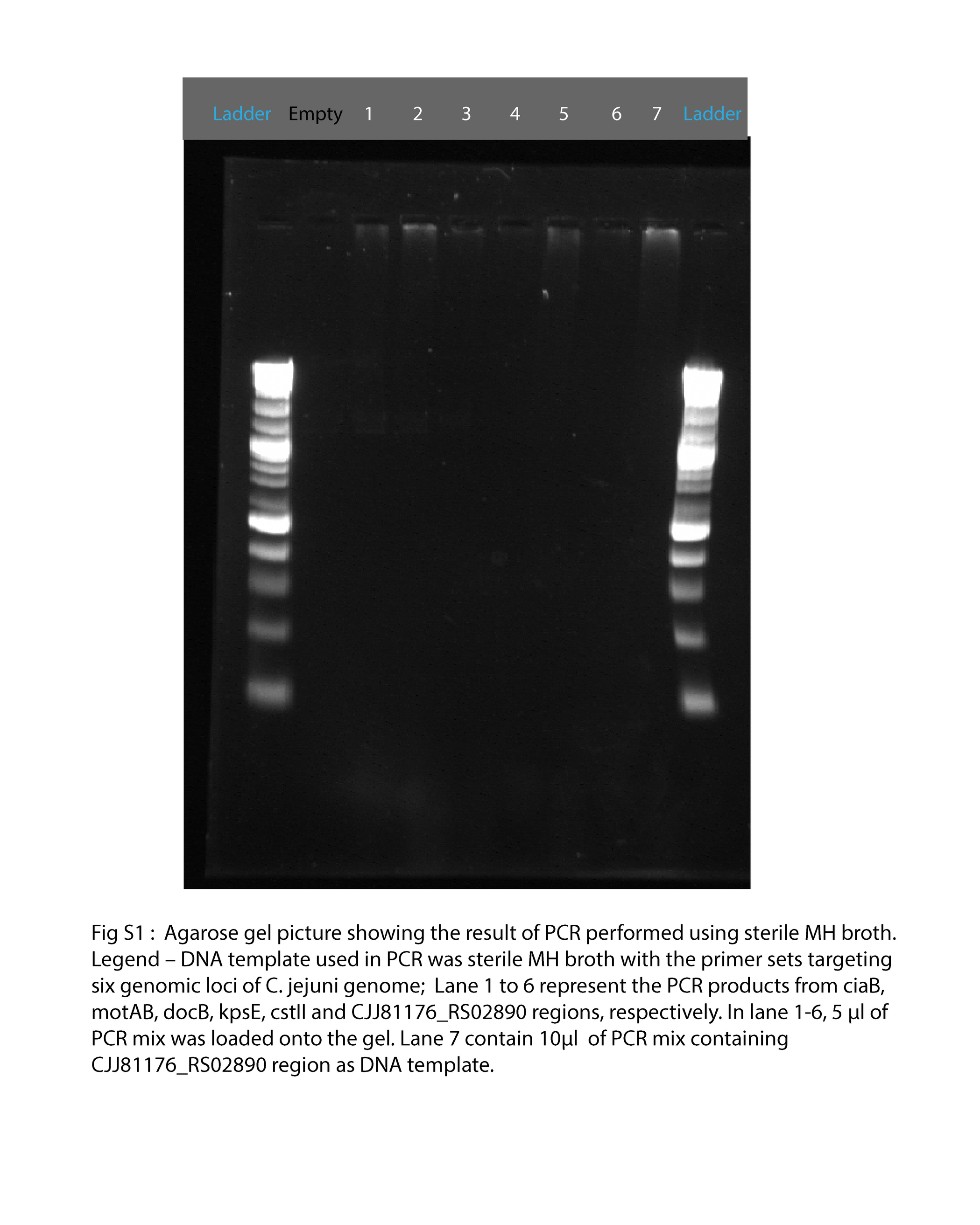

Supplement: S1 Fig — (TIF) [file pone.0241058.s001.tif]
