# Supplementary material for: Horizontal genetic exchange of chromosomally encoded markers between Campylobacter jejuni cells
Source: PLoS One. 2020 Oct 26;15(10):e0241058. doi: 10.1371/journal.pone.0241058 (PMC7588059; doi:10.1371/journal.pone.0241058)
Supplement: S1 Raw images — (PDF) [file pone.0241058.s002.pdf]

Image 1- Agarose Gel used in Figure 4 (A)

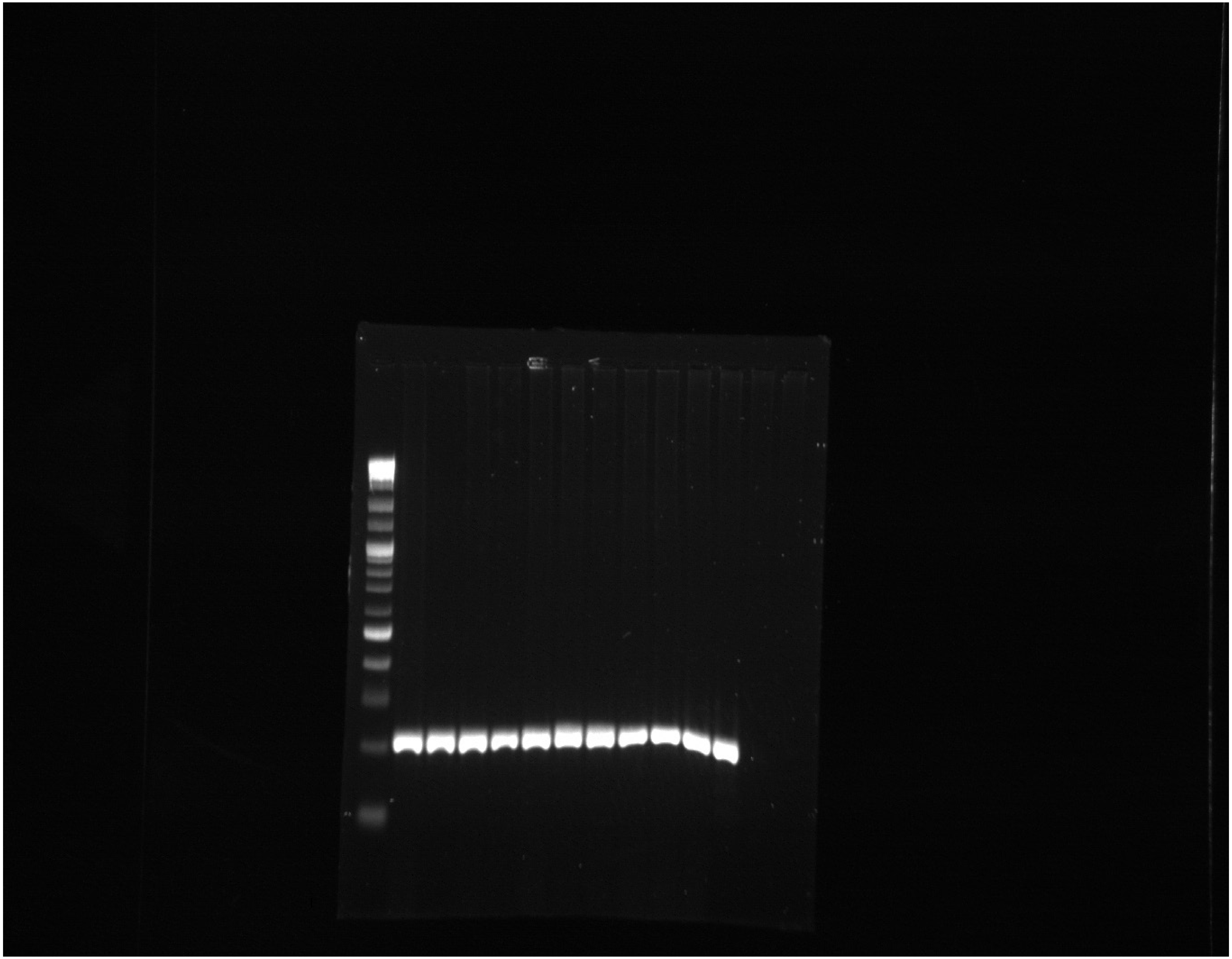

Ladder 1 2 3 4 5 6 7 8 9 10 11 12 13

Image 2- Agarose Gel used in Figure 4 (B)

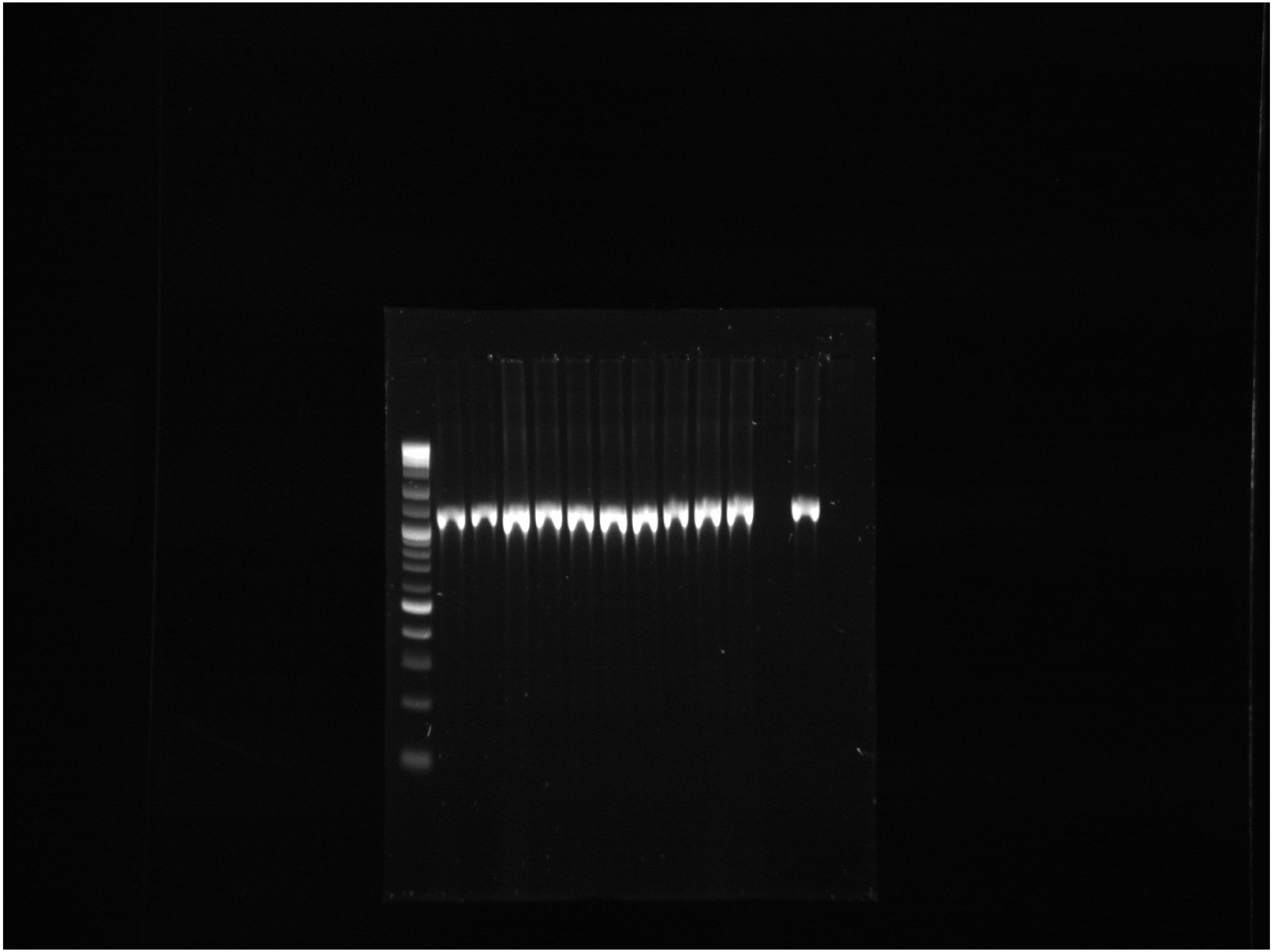

Ladder 1 2 3 4 5 6 7 8 9 10 11 12 13

Image 3- Agarose Gel used in Figure 10.

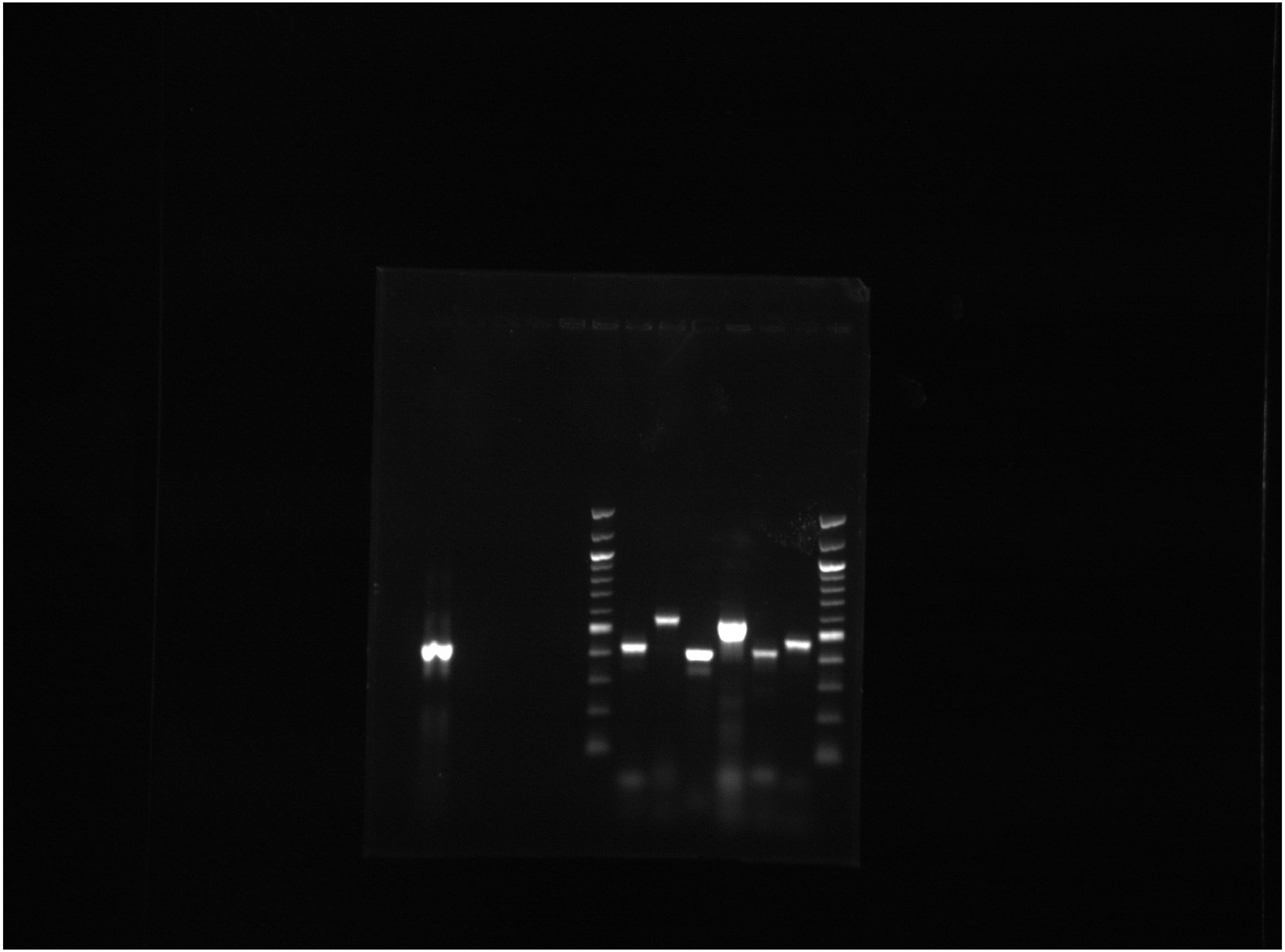

X X X X X X 1 2 3 4 5 6 7 8

Image 4 - Agarose gel used in Figure S1 (supplymentary information).

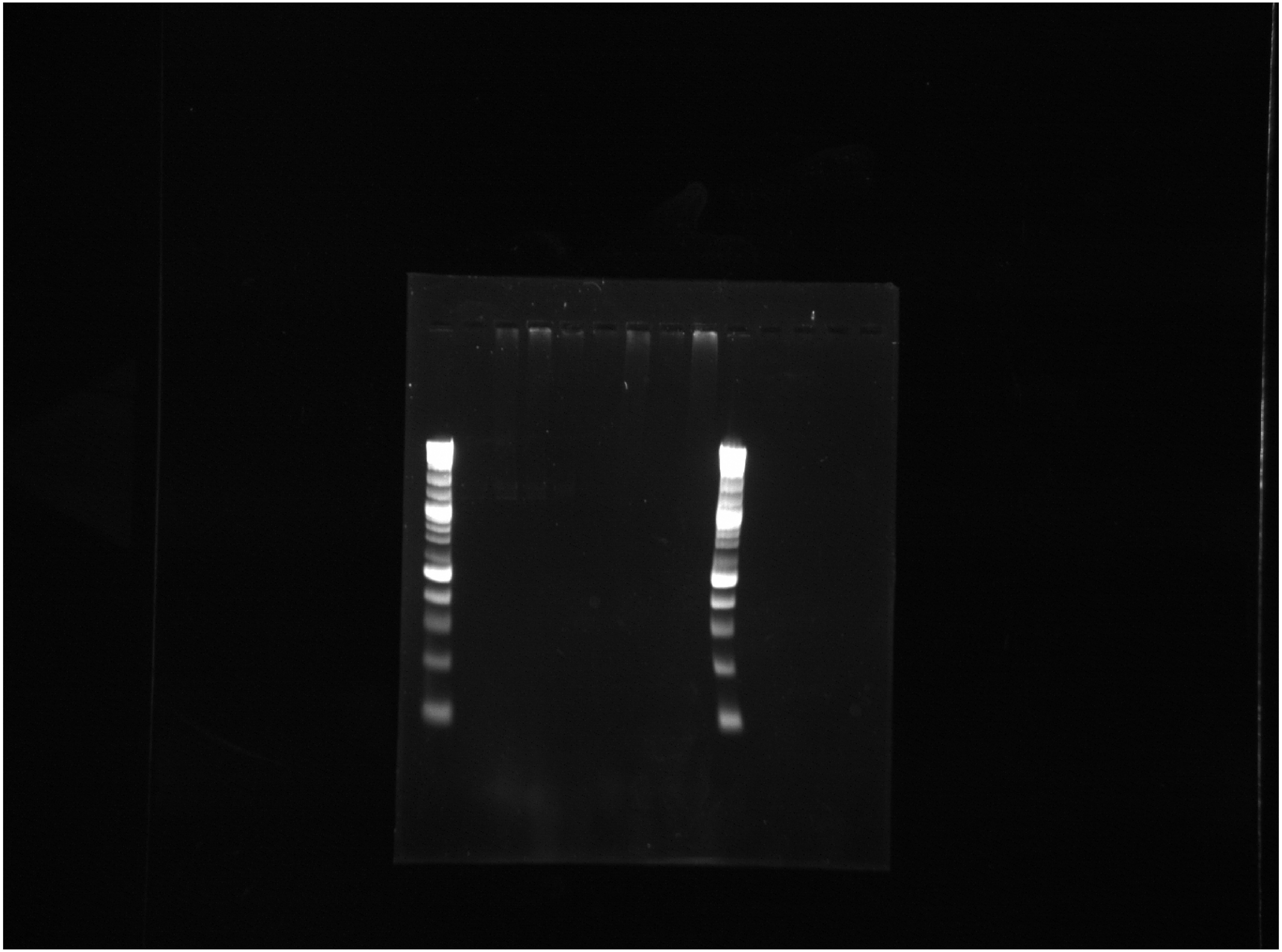

Ladder X 1 2 3 4 5 6 7 Ladder X X X X
